# Molluscicidal activities of curcumin-nisin polylactic acid nanoparticle on *Biomphalaria pfeifferi*

**DOI:** 10.1371/journal.pntd.0005855

**Published:** 2017-08-23

**Authors:** Michael E. Omobhude, Olajumoke A. Morenikeji, Oyetunde T. Oyeyemi

**Affiliations:** 1 Department of Zoology, University of Ibadan, Ibadan, Nigeria; 2 Department of Biological Sciences, University of Medical Sciences, Ondo, Nigeria; Sichuan University, CHINA

## Abstract

**Background:**

Snail intermediate host control is a widely canvassed strategy for schistosomiasis control in endemic countries. While there have been increasing studies on the search for potent molluscicides in the past years, the use of nanoparticulate agents as molluscicides is yet to gain wide attention. The aim of this study was to assess the molluscicidal potential of curcumin-nisin poly lactic acid (PLA) entrapped nanoparticle (CurNisNp) against *Biomphalaria pfeifferi*, a snail intermediate host for *Schistosoma mansoni*.

**Methodology/Principal findings:**

CurNisNp formulated by double emulsion method was tested against the young adults, < 1 week, 1-2-week old juveniles, 1 day (blastula) and 7 day-old (hippo-stage) egg masses of *B*. *pfeifferi*. Mortality in the different stages was determined after 96-h of exposure at varying concentrations (350, 175, 87.5, 43.75 and 21.88 ppm). The sub-lethal effects of CurNisNp on the hatchability of the 7-day-old egg masses and egg laying capacity of the young adult snails were determined. The CurNisNp diameter, polydispersity index (PDI), zeta potential and drug entrapment efficiency were 284.0 ± 17.9 nm, 0.166 ± 0.03, -16.6 ± 2.45 mV and 35.0% respectively. The < 1 week old juveniles and the 1-day-old egg stage (blastula) of *B*. *pfeifferi* with LC_50_ 277.9 ppm and 4279.5 ppm were the most susceptible and resistant stages to the drug respectively. CurNisNp was also observed to cause significant reductions (P<0.05) in egg hatchability and egg laying capacity with strong negative correlation between egg laying capacity and concentration (r = -0.928; P<0.05).

**Conclusion/Significance:**

This study showed that CurNisNp has molluscicidal activities on different developmental stages of *B*. *pfeifferi*. It is therefore recommended that the formulation be more optimised to give a nanoparticle with a narrow range monodispersed PDI for better drug distribution and eventual greater molluscicidal activities.

## Author summary

Elimination of snail intermediate host of schistosomiasis has been widely advocated as an arm of integrated control for schistosomiasis. This becomes important as reports now abound on development of praziquantel resistance in schistosomes. Nanotechnology has been applied in control of tropical diseases including schistosomiasis. The few available nanomedicines were targeted against the *Schistosoma* worms without research efforts on the use of the same technology against the molluscan host of the parasite. This study seeks to assess the molluscicidal potential of CurNisNp on *Biomphalaria pfeifferi*; a snail intermediate host of *Schistosoma mansoni*. The formulation was tested on the different developmental stages of the snail. The formulation showed decreasing molluscicidal activity with increase in age of the snail. Impairment of the egg laying capacity of the adult snails was related to nanoparticle concentration. The egg was the most resistant stage. It is evident from the study that the formulated nanoparticle had molluscicidal properties and to further harness this potential, optimisation of the formulation to give a narrow range monodispersed polydispersity index for better drug distribution is recommended.

## Introduction

Trematodes, the causal agents of schistosomiasis and fascioliasis are important parasites of economic and public health implications in most of sub-Saharan Africa. Schistosomiasis affects over 240 million people worldwide, with up to 700 million individuals living at risk of infection [1]. The disease caused up to 250,000 deaths per year in the last decade [2]. Chemotherapy has been the most adopted means of control of schistosomiasis in developing countries. The increase in number of individuals that need to be treated with praziquantel (PZQ) necessitates the corresponding increase in PZQ deployment in sub-Saharan Africa thus raising concerns about the emergence and establishment of *Schistosoma* resistance to PZQ [3].

The control of snail intermediate hosts in an attempt to break the parasite transmission cycle is a widely advocated strategy in schistosomiasis control. Niclosamide, a chemical molluscicide has recorded success in this regard, but its toxicity against non-targeted organisms has been a major set-back to its general adoption [4].

Intensive studies have been conducted in search of agents which are more environmentally friendly to combat the intermediate hosts of *Schistosoma*. Efforts are directed particularly towards molluscicides of plant origin with several studies reported across the world [5–11]. Nevertheless, there is currently no licensed plant-derived molluscicide despite this myriad of studies. This could be due to inability to standardize these findings for wide scale use. The feasibility of plant-induced toxicity on non-targeted organisms cannot also be ruled out.

Nanotechnology has gained increasing interest in biomedicine with the utmost aim of effective delivery of bioactive agents. Particularly it has been widely applied to combat parasitic agents [12,13]. While attention is often drawn towards *Schistosoma* parasites [14,15], little is known about the application of nanomedicine against the snail intermediate hosts of the parasites. Curcumin and nisin are naturally derived non-toxic compounds [16,17] with a wide range of activities [18–21]. The two compounds have been shown to have antibacterial, anti-inflammatory and anticancer properties [18,19]. Curcumin has been reported to be efficacious against adult *Schistosoma mansoni* [20,21]. The higher bioavailability of these compounds in nanoparticulate forms and improved efficacy against some biological agents [22,23] could make their combination into a nanoparticulate formulation a desirable molluscicidal agent. The aim of this study was therefore to evaluate the molluscicidal potential of curcumin-nisin PLA entrapped nanoparticle on *Biomphalaria pfeifferi*, a snail intermediate host of intestinal schistosomes.

## Materials and methods

### Ethics statement

The institutional animal care and use committee in our Nigerian institutes granted waiver since freshwater snails are not among the selected animals that approval is needed. Also, in Nigeria there are no agencies that issue permit for collection of wild freshwater snails. So, no permit was obtained.

### Drug

The test substance (drug): curcumin-nisin poly-lactic acid nanoparticles (CurNisNp), is a yellow biodegradable hygroscopic powder of 35.0% composition by mass of the active ingredients. It was prepared by the double emulsion-diffusion-evaporation method at the National Institute of Immunology, New Delhi, India.

### Preparation of curcumin-nisin poly-lactic acid nanoparticle (CurNisNp)

The formulation was prepared by the double emulsion-diffusion-evaporation method. Curcumin and nisin of equal amount (5 mg) was subjected to dissolution in 200 μL 1% polyvinyl alcohol (PVA). The mixture was dispensed into 50 mg of poly lactic acid containing organic solvents and was sonicated for 1 min to obtain a primary emulsion. The emulsion was added dropwise to 16 mL 2% PVA containing 1% sucrose. The secondary emulsion formed was sonicated at 30 W, 40% duty cycle for 3 mins to form a nanosuspension. This was continuously stirred until all the solvents were evaporated. The nanosuspension was subjected to ultracentrifugation (16,000 rpm for 15 min) and then washed. The washing was repeated two times and the formulation was lyophilized with 5% mannitol as cryoprotectant.

### Characterization and drug entrapment efficiency of CurNisNp

The physical properties of the formulation including the size, zeta potential and polydispersity index (PDI) were measured by dynamic light scattering method using Zetasizer Nano-ZS (Malvern Instruments, UK). The size and PDI of the nanoparticle were determined by dispersing a homogenous solution of the formulation in sizing cuvette and then measured by Zetasizer Nano-ZS. Clear zeta cell was used for zeta potential analysis. Drug encapsulation efficiency was determined by a modified method described by Dauda et al. [13].

### *In vitro* release kinetics of drug-entrapped nanoparticle

Ten milligram (10 mg) of Cur-Nis-NP was dissolved in 10 mL PBS (140 mM NaCl, 10 mM phosphate buffer, 3 mM KCl, pH 7.4) [21]. The homogenous solution was incubated in a rotary shaker at 200 g. The sample was centrifuged at 16,000 g for 10 min at specific time after which 1 mL of supernatant was withdrawn and then replaced with 1 mL of fresh PBS [13]. Curcumin and nisin (2.5 mg each) was dissolved in 5 mL methanol to form a stock solution (100 μg/mL). The working standard concentrations (5–70 μg/mL) were prepared from the stock with PBS. The UV-absorbance was measured at 290 nm. The UV-absorbance analysis of supernatant from curcumin-nisin PLA entrapped nanoparticle was carried out at different time intervals. The *in vitro* drug release from the formulated nanoparticle was estimated from the standard plot obtained from UV-absorbance analysis of free curcumin-nisin.

### Snail collection

Adults of *Biomphalaria pfeifferi* were collected from Odo Ona River (latitude 7°21ʹ-7°22ʹN; longitude 3°50ʹ-3°51ʹE) in Ibadan, Oyo State, Nigeria. They were properly washed in water and transferred into plastic containers with good ventilation. The snails were brought to the Parasitology Research Laboratory of the Department of Zoology, University of Ibadan for further analysis. Snails were collected blinded of their infection status and were later subjected to cercariae screening through exposure to sunlight for 1–2 h in dechlorinated tap water. Only clean snails were used for the study.

### Snail culture

Twenty five (25) adult *B*. *pfeifferi* were transferred into a culture jar (aquarium) lined with a transparent polythene bag containing dechlorinated tap water. The snails were fed with blanched dried lettuce (*Lactuca sativa*), and CaCO_3_ pellets were used as calcium supplements. They were maintained at room temperature (26–29°C) under natural light:dark cycles. The egg masses laid by snails were cut out with a scalpel and transferred into a petri dish containing dechlorinated tap water. Incubation was done as previously described [8,24]. The snails hatched within 6-7-days of incubation, and were subsequently transferred and maintained in a larger container to accommodate their growth.

### Molluscicidal bioassay activity test

The molluscicidal bioassay activity tests were carried out on the snail developmental stages (<1 week old juveniles, 1–2 weeks old juveniles, and 5–6 weeks old young adults) in line with the WHO guidelines [25,26]. Ten (n = 10) snails were placed in each test container for all the stages tested except the < 1 week old *B*. *pfeifferi* juveniles where number of snails exposed was n = 22. The snails at different developmental stages were placed in 40 mL of varying concentrations (350 ppm, 175 ppm, 87.5 ppm, 43.75 ppm and 21.88 ppm diluted with dechlorinated water) of the nanoparticle formulation and mortality was observed after 96-h exposure. Snails’ avoidance or protective behaviours during exposure were observed. Observation and examination for mortality were done using hand lens or dissecting microscope where necessary. The snails that could move or with an active heart beat (as observed under the microscope) were counted as living and vice versa. The percentage mortality was calculated.

### Ovicidal activity and egg hatchability

The ovicidal bioassay activity and egg hatchability tests were carried out on the egg masses of uninfected adult *B*. *pfeifferi* using 1 day old blastula stage and 6–7 days old pre-hatched hippo- stage respectively in line with the methods [27,28]. Two to three egg masses (adding up to an average of 26 embryos) were harvested from the snail cultures and placed in each test container containing different concentrations of the test material. The egg masses were observed every 24 h for one week and afterwards biweekly for four weeks at room temperature and normal diurnal lightening. After every 24 h, the snail egg masses were examined under the microscope for viability and then the percentage mortality calculated. At the pre-hatched stage, the snail embryos were observed under the microscope for movement within their gelatinous egg masses. The pre-hatched eggs were further examined for number of embryos hatched. The egg hatchability was calculated as percentage difference relative to the total number of eggs exposed.

### Egg laying capacity determination

The egg laying capacity of young adult *B*. *pfeifferi* snails was determined by maintaining and monitoring snails’ oviposition to assess their reproductive viability daily for 5 days post exposure to CurNisNp. This was achieved by counting the number of egg masses laid by the different groups of young adult snails exposed [27,29]. The total number of eggs laid by treated and control groups of snails were estimated.

All experiments were performed in duplicate with values expressed as mean ±SD. The negative control groups were placed in dechlorinated water.

### Statistical analysis

The data were subjected to SPSS version 21 for windows for analysis. Two-way ANOVA was used to test significant differences in snail mortality in different concentrations. Probit regression graphing was used to determine the LC_50_ and LC_90_ of the nanoparticulate formulation_._ Linear Regression analysis and Pearson’s correlation were applied to determine the relationship between snail mortality/egg hatchability/egg laying capacity (fecundity) and test concentrations. *P* <0.05 was considered statistically significant.

## Results

### Properties of nanoparticle

**Fig 1:**
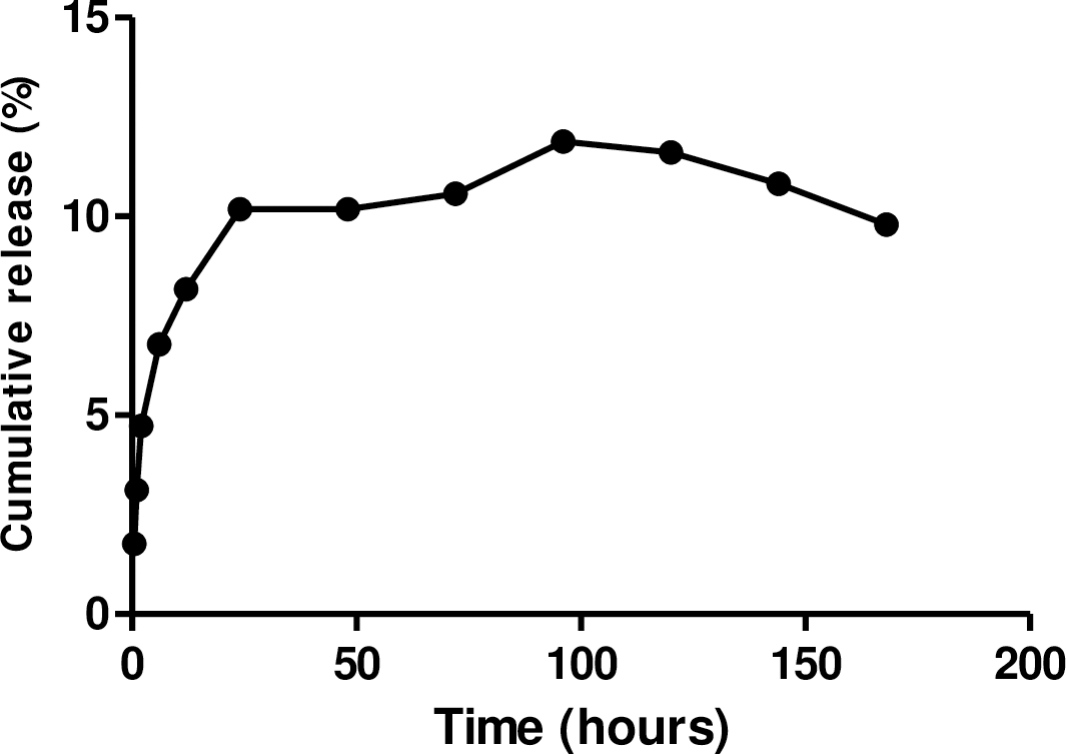
*In vitro* release of Cur-Nis in formulated nanoparticle.

The CurNisNp diameter, PDI, zeta potential and drug entrapment efficiency were 284.0 ± 17.9 nm, 0.166 ± 0.03, -16.6 ± 2.45 mV and 35.0% respectively. The *in vitro* release of Cur-Nis is presented in Fig 1.

### Snail behaviour and mortality

**Fig 2:**
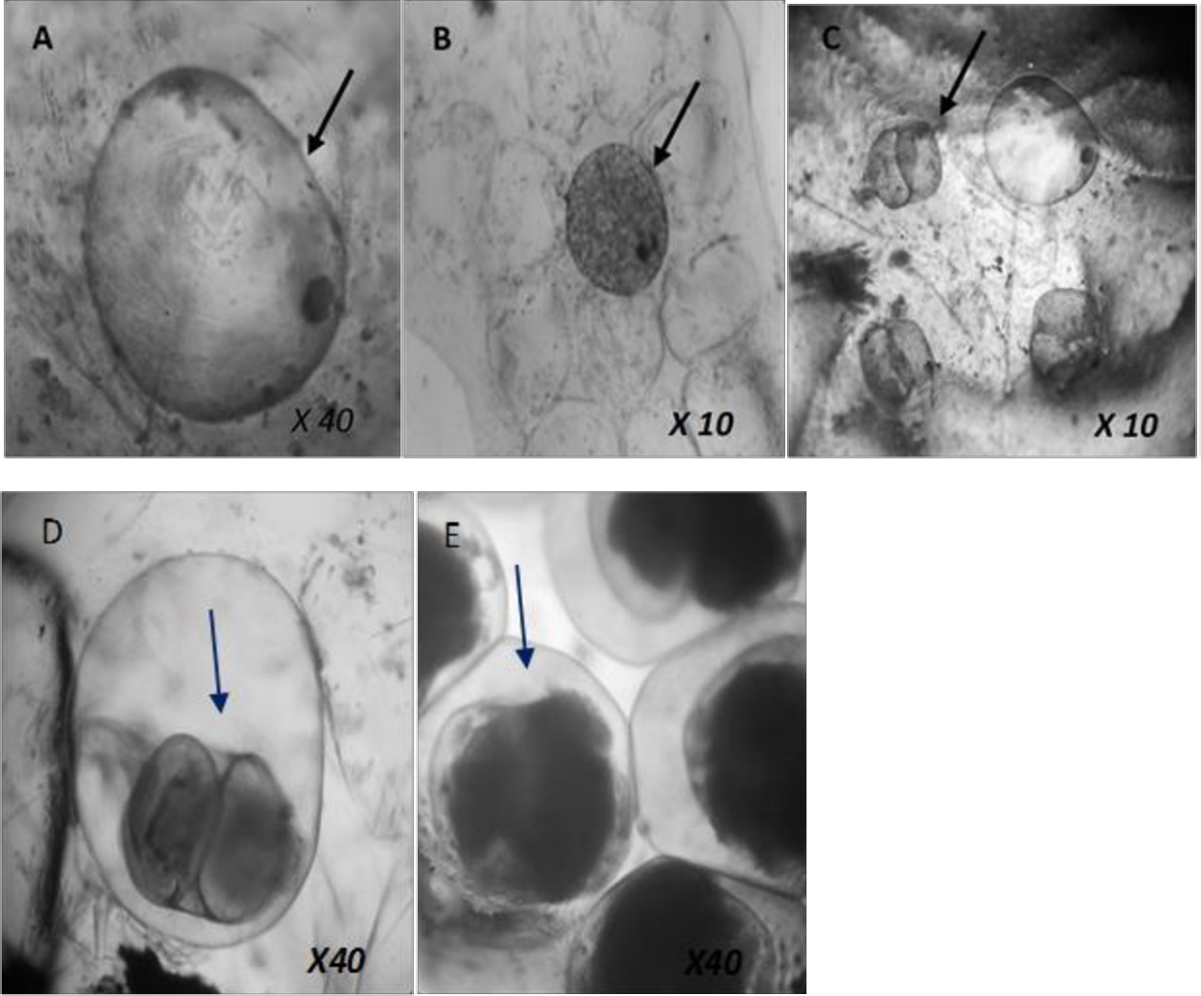
Photomicrographs showing dead/deformed and normal *B*. *pfeifferi* embryos.

The protective behaviors of the snails following their introduction into the test concentrations included crawling along the walls of the containers, surfacing behavior and partial retraction of their head-foot. Normal crawling activities resumed after only a few minutes. Mortality in snails was not dependent on concentrations of the formulation (P>0.05). However, mortality was significantly higher in the tested concentrations compared with the negative control (P<0.05). The formulation killed more than half of the young adult snails (5–6 week-old) (60.0–70.0%) in concentrations 43.75, 87.5 and 350.0 ppm. The <1 week old juvenile of *B*. *pfeifferi* were the most susceptible to CurNisNp with mortality ranging from 82.2–100.0 ppm (Table 1). The 1 day exposed egg masses were the least susceptible group with highest mortality of embryos recorded in 175.0 ppm of CurNisNp. No embryonic death was recorded in 7-day-old egg masses exposed to 175.0 ppm and 43.75 ppm of the nano-formulated drug. Half of the 1-2-week-old juveniles (50.0%) of *B*. *pfeifferi* died at 350.0 ppm after 96-h exposure (Table 1). The <1 week-old juvenile snails had the lowest LC_50_ (277.9 ppm) and LC_90_ (676.4 ppm) while the 1-day-old egg had the highest LC_50_ (4279.5 ppm) and LC_90_ (8184.6 ppm) (Table 2). The photomicrographs of toxicity effects of CurNisNp on *B*. *pfeifferi* embryos are presented in Fig 2A–2E (A; dead embryo viewed 1 week after exposure, B; dead embryo viewed 4 weeks after exposure, C; empty shell of dead prehatched stage embryo beside a dead blastula stage embryo viewed 4 weeks after exposure, D; deformed embryo, E; normal embryo at prehatched stage).

**Table 1:** Percentage mortality (± SD) of freshwater snails at different developmental stages.

		Concentration (ppm)					
Age	Stage	350.0	175.0	87.5	43.75	21.88	Control
1 day	Egg (blastula)	4.9 ±2.12	20.0 ± 2.83	3.2 ± 1.41	6.5 ±0.00	3.2 ± 1.41	0.0
7 days	Egg (hippo)	25.6 ±7.78	0.0 ± 0.0	40.5 ± 6.36	0.0 ± 0.0	47.5 ± 20.50	0.0
<1 week	Juvenile	100.0 ±8.49	97.7 ± 2.12	88.6 ± 3.54	82. 2 ± 2.12	93.3 ± 19.80	0.0
1–2 weeks	Juvenile	50.0 ±1.41	45.0 ± 2.12	25 ± 0.71	20.0 ± 1.41	25 ± 0.71	0.0
5–6 weeks	Adult	70.0 ±0.00	20 ± 0.00	70 ± 0.00	60.0 ± 0.00	50 ± 1.41	0.0

**Table 2:** Probit analysis of lethal concentrations of CurNisNp against *B*. *pfeifferi* at different stages exposed.

				Lethal concentration (ppm) (95% CI)	
Age	Stage	Regression equation	R^2^	LC_50_	LC_90_
1 day	Egg (blastula)	y = 0.0001x + 0.062	0.037	4279.5 (2645.79–6769.2)	8184.6 (7476.23–109124.5)
7 days	Egg (hippo)	y = -0.0002x + 0.247	0.022	1072.7 (872.33–1326.2)	2767.7 (1927.213–3623.2)
<1 week old	Juvenile	y = 0.0004x + 0.867	0.557	277.9 (210.45–344.5)	676.4 (510.45–724.6)
1–2 weeks	Juvenile	y = 0.0009x+0.204	0.840	318.9 (245.12–398.3)	750.0 (635.89–857.2)
5–6 weeks	Adult	y = 0.0002x + 0.433	0.025	339.1 (229.7965–448.2)	2373.4 (1472.34–3258.5)

### Effects of nanoparticle on snail hatchability

The hatchablity of snails was significantly higher in the negative control than in the exposed groups (P<0.05). The embryos hatching from the gelatinous masses significantly increased with time (P<0.05). No snail was hatched out in the nanoparticulate concentrations 350.0, 175.0 and 87.5 ppm after 24-h exposure. All snails had hatched after 144-h exposure in concentrations 175.0 and 43.75 ppm (Table 3). Snail hatchability was independent of nanoparticle concentration (P>0.05); however, all the hatched snails died within a short period compared with the negative control.

**Table 3:** Percentage hatchability (± SD) of *B*. *pfeifferi* eggs exposed to CurNisNp at the pre-hatched stage.

	Time (hours)					
Conc. (ppm)	24	48	72	96	120	144
350.0	0.0 ± 0.0	7.0 ± 0.7	23.3 ± 1.4	46.5 ± 2.8	74.4 ± 8.5	74.4 ± 8.5
175.0	0.0 ± 0.0	2.3 ± 0.7	27.3 ± 0.7	72.7 ± 2.8	93.2 ± 2.1	100.0 ± 1.4
87.5	0.0 ± 0.0	0.0 ±0.0	5.8 ± 2.12	23.1 ± 1.41	34.6 ± 1.4	67.3 ± 0.7
43.75	4.4 ± 1.4	8.9 ± 0.0	33.3 ± 5.0	48.9 ± 5.7	64.4 ± 3.5	100.0 ± 0.7
21.88	3.4 ± 1.4	8.5 ± 3.5	13.6 ± 5.7	33.9 ± 14.1	47.5 ± 19.8	50.8 ± 21.2
0.0	89.1 ± 121.4	100.0 ± 145.0	100.0 ± 126.2	100.0 ± 101.6	100.0 ± 101.3	100.0 ± 83.8

### Effects of nanoparticles on snail fecundity

The egg laying capacity of the young adult snails exposed to CurNisNp was significantly lower than in the negative control (P<0.05). The fecundity rate was also concentration dependent (P<0.05). The young adult snails exposed to 21.88 ppm had the highest egg laying capacity (48.5 ± 2.91) while those exposed to 350.0 ppm showed the lowest egg laying capacity (14.5 ± 4.23) (Table 4). The Pearson correlation showed significant inverse relationship between CurNisNp concentrations and egg laying capacity of the snails (r = -0.928; P<0.05). The average fecundity rate however showed no significant differences with days of exposure of snails (P>0.05).

**Table 4:** Egg laying capacity (± SD) of snails exposed to CurNisNp.

Conc. (ppm)	Day 1	Day 2	Day 3	Day 4	Day 5	Total no. of eggs/group
350.00	0.0 ± 0.0	3.0 ± 4.24	5.0 ± 7.07	1.5 ± 2.12	5.0 ± 7.07	14.5 ± 4.23
175.00	5.0 ± 7.07	3.0 ± 4.24	5.0 ± 7.07	8.5 ± 2.12	4.0 ± 5.66	26.0 ± 4.58
87.50	0.0 ± 0.0	8.0 ± 2.83	7.0 ± 1.41	3.5 ± 4.95	11.0 ± 1.41	29.5 ± 4.48
43.75	7.0 ± 1.41	8.0 ± 2.83	6.0 ± 0.00	12.5 ± 0.71	7.5 ± 3.54	41.0 ± 2.86
21.88	11.0 ± 1.41	6.0 ± 0.00	11.0 ± 1.41	10.0 ± 5.66	10.5 ± 2.12	48.5 ± 2.91
0.0	13.5 ±0.71	15.0 ± 1.41	19.0 ± 1.41	23.0 ± 7.07	25.0 ± 4.24	95.5 ± 5.47

## Discussion

The curcumin-nisin PLA entrapped polymeric nanoparticle used in this study is novel and could serve as an ideal molluscicide. The PDI of the formulation will facilitate moderate distribution [30] and therefore optimisation of the drug formulation to give a narrow range monodispersed PDI for better drug distribution within the target organism is recommended. This is achievable by varying the concentrations of surfactant, organic and aqueous phase, and drug-polymer ratio [13]. In addition, the origin of the drug, as a formulation from nisin and curcumin, suggests that it will exhibit low to zero toxicity. There is presently no study on toxicity of nisin on non-target organisms, but one study has shown safety of extract from *Curcuma longa* (the parent plant from which curcumin is obtained) on brine shrimps [31].

The snails’ avoidance behaviors following exposure to the test concentrations of the nanoparticulate drug is an indication of possible molluscicidal effects. These observations are in line with those of many Nigerian workers [8,9,32,33] and workers elsewhere [34–36]. The observed crawling out (distress syndrome) from the test concentrations and aggregation at the water-air interface by the exposed snails was taken as an escape or avoidance behavior which has been described by the aforementioned workers. This behavior which is as a result of response to loss of water balance [37] helps to increase their chances of survival and as a result hinder the action of molluscicides [10].

Susceptibility of *B*. *pfeifferi* to the CurNisNp was dependent on snail developmental stages. This kind of developmental stage-dependent variation in susceptibility to a molluscicidal agent has also been observed in a previous study [8]. The lack of association between snail mortality and nanoformulation concentration contradicts the findings of other studies [8,32,38–40]. This was particularly observed in those instances where the nanoparticulate drug showed higher activity at low doses when compared with high doses. This contrast with other studies could have been due to the ability of the formulation to penetrate membrane barriers in the organism to reach the target tissues or organ, even at lower doses. The use of nanoparticle formulations may confer an advantage over the use of unbound drug or plant extracts, as employed in the aforementioned studies, as lower doses of nanoparticles could exert similar efficacy. The lower LCs values recorded in the juveniles indicates their higher susceptibility to CurNisNp, but the relatively higher value recorded for the young adult snails could have been due to increase resistance to the formulation as the snails advanced in age. This is reasonable as tolerance to adverse environmental conditions increases with the age-dependent acquisition of better developed organs e.g. mantle and periostracum. Our investigation did not show the concentration-dependent nature of molluscicidal action on embryos reported in earlier studies [8,41]. However, our study shared similar morphological alterations such as deformation of gastrula with other studies [8,39].

Reduction in snail hatchability in nanoparticle exposed groups suggests an increase in bioavailability which ensured delivery of PLA encapsulated drug to the targeted pre-hatched snails within the protective gelatinous egg masses. Nanoparticles are known for their ability to penetrate host barriers [42] and CurNisNp may be no exception to this. Another possibility is that increase in CurNisNp lipophilicity may facilitate its penetration of the snail eggs, thus leading to greater inhibition of hatching in drug exposed groups, when compared with the negative control. Prolonged time of exposure could however undermine reduction in eggs hatching. The death of all the hatched juvenile snails in nanoparticulate exposed groups compared with the negative control group suggests the cumulative effects of the formulation during exposure. The consistent release of the nanoparticulate drug for more than 5-day (120 h) exposure period could be responsible for this cumulative effect.

Although this is the first report on reproductive toxicity of curcumin-nisin nanoparticle in freshwater snails, metallic-nanoparticle induced reproductive toxicity has been observed in molluscs [43], crustaceans [44,45], and marine invertebrates [46]. The snail fecundity rate reduction observed in the formulation exposed groups in our study was similar to observations in some molluscicides and anti-parasitic agents [29,47–49]. The observed reduction in snail egg production might have been due to metabolic changes possibly caused by prolonged exposure of the snail to CurNisNp, which might include destruction of gametogenic cells and damage of hermaphrodite glands possibly resulting from decrease in tissue proteins, DNA damage (apoptosis), or degeneration of cells of these vital organs [29,50,51]. Such a feature is most desirable in a molluscicide of the kind tested here, which is not strongly toxic against the adult snails, as it could be effective in regulating snail populations without necessarily compromising the functional role of the adult snails within the aquatic ecosystem.

## Conclusion

It is clear from this study that CurNisNp is a potential molluscicide. It is active against all the snail stages, but with different dynamics of potency. Although the formulation may not prevent hatching of juvenile snails from the egg masses at the pre-hatched stage, it significantly reduced the number of viable juveniles. The adult snails were relatively resistant to the molluscicide, but the significant reduction in their egg laying capacity makes the formulation a potential desirable molluscicide. It is therefore recommended that the formulation be more optimised to give a nanoparticle with a narrow range monodispersed PDI for better drug distribution and eventual molluscicidal activities. More studies on toxicity, stability and photosensitivity of the nanoparticle should also be considered.
